# Supplementary figures and images for: Multi‐Omic Associations of Epigenetic Age Acceleration Are Heterogeneously Shaped by Genetic and Environmental Influences
Source: Aging Cell. 2025 May 5;24(8):e70088. doi: 10.1111/acel.70088 (PMC12341791; doi:10.1111/acel.70088)

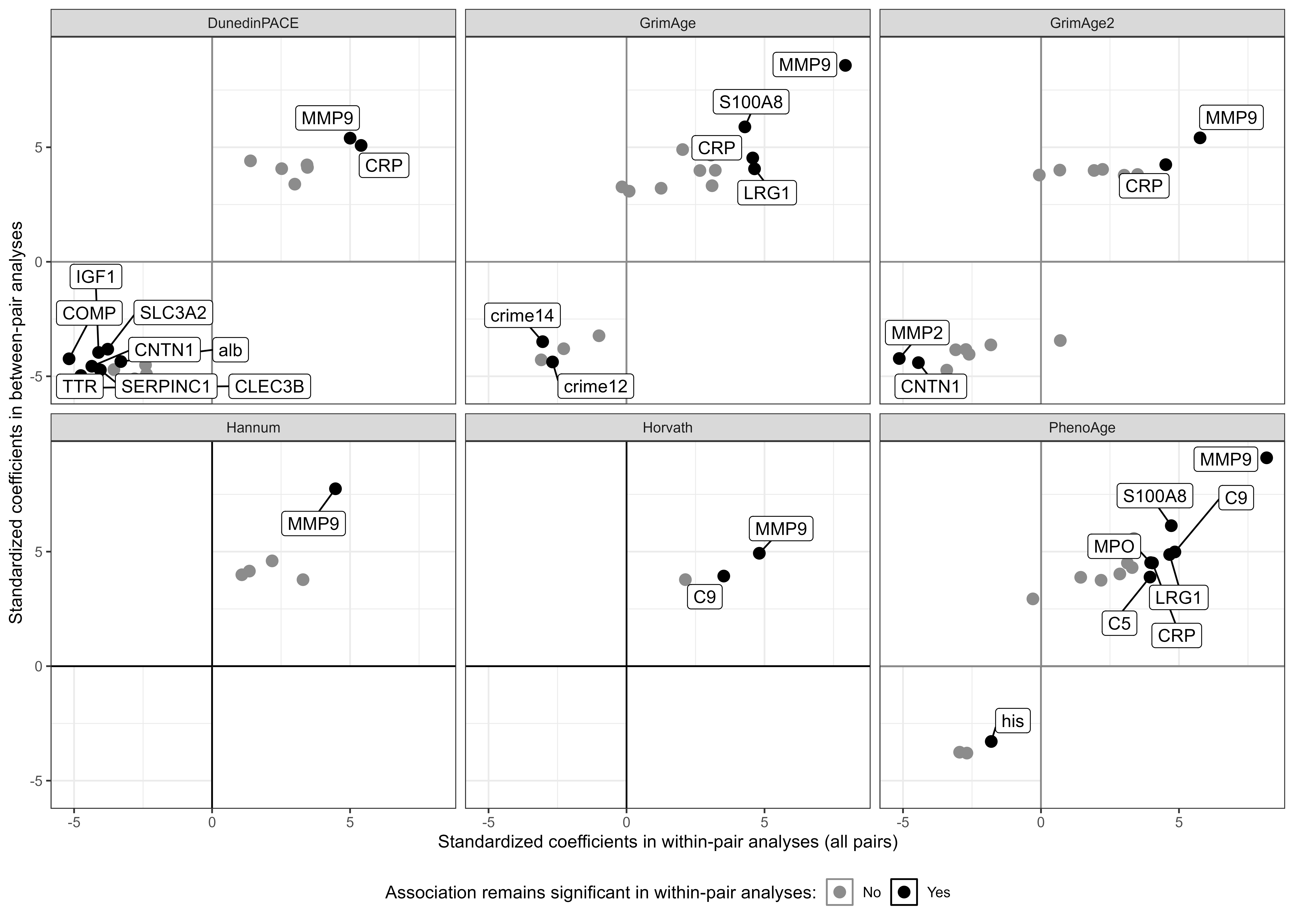

Supplement: Supplementary file 1 — Figure S1. Scatter plot of standardized coefficients in both between‐pair and within‐pair analyses in FinnTwin12. [file ACEL-24-e70088-s001.png]
